# Successful Long-Term Use of Eltrombopag in a Patient with Refractory Severe Thrombocytopenia Associated with Chronic Lymphocytic Leukemia That Allowed Oral Anticoagulant Treatment for Severe Cardiomyopathy

**DOI:** 10.1155/2017/9538920

**Published:** 2017-04-02

**Authors:** Juárez Salcedo Luis Miguel, Gil-Fernández Juan José

**Affiliations:** Division of Hematology, Principe de Asturias University Hospital, Madrid, Spain

## Abstract

Autoimmune cytopenias (AICs) are frequently associated with chronic lymphocytic leukemia (CLL). The most common of these AICs is autoimmune hemolytic anemia (AIHA); the second most is immune thrombocytopenia (ITP). Here, we report on a patient with CLL-associated ITP, with thrombocytopenia refractory to corticosteroids and intravenous immunoglobulins, in which continuous oral treatment with Eltrombopag allowed initiation and maintenance of an oral anticoagulation treatment with Acenocoumarol that was indicated because of a severe arrhythmogenic cardiomyopathy.

## 1. Introduction

While other autoimmune disorders have been reported in CLL, autoimmune cytopenias (AICs) are by far the most frequent immune complications (4–10% of total cases) [1–3]. CLL patients have a greater risk of developing this phenomenon, almost entirely related to blood cells in the form of autoimmune hemolytic anemia (AIHA), immune thrombocytopenia (ITP), pure red cell aplasia (PRCA) and, more rarely, autoimmune granulocytopenia (AIG) [2]. Among CLL patients that develop an AIC, the relative frequency of these AICs is as follows: AIHA (55–70%), ITP (18–47%), and PCRA and AIG, at 12% and 4%, respectively [1, 3].

ITP occurs in 2–5% of all CLL patients, although it may be underestimated unless a bone marrow examination is performed in all patients with thrombocytopenia [4]. In CLL patients, thrombocytopenia produced by multiple mechanisms (lymphoid bone marrow infiltration, hypersplenism, and antiplatelets autoantibodies) can be clinically and biologically relevant.

The mechanism of ITP is similar to that of AIHA as an antibody-mediated process. However, ITP is less well understood than AIHA as key experiments are not usually performed in ITP. The pathogenic autoantibody is not easy to detect on platelets and less is known about isotype [5].

The goal of ITP treatment is to prevent or treat bleeding complications. Among the therapeutic arsenal, the second-generation agonists of the thrombopoietin (TPO) receptor, Romiplostim and Eltrombopag, have been introduced in clinical practice for the treatment of chronic ITP patients as a second-line therapy during the last 7 years [6, 7]. Currently, Eltrombopag has also been approved for the treatment of patients with Hepatitis C Virus-associated thrombocytopenia candidates to initiate an interferon-based antiviral treatment and for the treatment of acquired aplastic anemia refractory to immunosuppressive treatments.

Widely used for primary ITP, Eltrombopag has shown responses in CLL-associated ITP in retrospective case series [8]. Since TPO receptor agonists are not immunosuppressants, they have a low risk of infectious complications, which is also attractive in CLL patients [9].

We report the case of a patient with CLL and moderate thrombocytopenia refractory to corticosteroids and intravenous immunoglobulins, in which continuous oral treatment with Eltrombopag has allowed us to initiate and maintain an oral anticoagulant treatment with Acenocoumarol that was indicated due to a severe arrhythmogenic cardiomyopathy.

## 2. Case Report

A 69-year-old male patient with a personal history of hypertension, type 2 diabetes, and chronic auricular fibrillation under treatment with Propafenone since January 2003 but without ventricular dysfunction in echocardiogram. He was diagnosed with a Binet A/Rai 0 stage CLL, with 13q14 deletion in November 2003, and remained stable without treatment until 2011.

In June 2011, the patient developed B symptoms and a CT scan revealed multiple abdominal lymphadenopathy and splenomegaly (17 cm), due to disease progression (Binet C/Rai IV stage) with Hb 9.95 g/dl, platelets 91.4 × 10^9^/L, and WBC 124 × 10^9^/L (90% lymphocytes). Three cycles of Bendamustine treatment as monotherapy (70 mg/m^2^/day) × 2 days every 28 days followed by 2 cycles of Bendamustine plus Rituximab were administered, achieving a partial response after treatment. In January 2012, treatment was interrupted due to poor hematologic tolerance and permanent cytopenias (Hb 11 g/dl, platelets 58 × 10^9^/L, WBC 2.01 × 10^9^/L, and neutrophils 0.91 × 10^9^/L). In the following weeks, hemoglobin levels and neutrophils were normalized, with remaining platelets under 50 × 10^9^/L. A bone marrow biopsy was performed; the report described areas of moderate infiltration by small and mature lymphocytes cells, with conserved numbers of megakaryocytes of heterogeneous aspect.

In this context, a diagnosis of immune thrombocytopenia (ITP) was made. The patient commenced oral prednisone at a dose of 1 mg/kg and he received 1 g/kg/day of intravenous immunoglobulins in two consecutive days. The platelet count remained < 40 × 10^9^/L despite the treatment but without signs of bleeding.

In October 2012, the patient required hospitalization due to symptoms and signs of congestive cardiac failure, with left ventricular ejection fraction of 25% by echocardiogram in addition to dilated myocardiopathy signs. Anticoagulant therapy for thromboembolic risk prevention was proposed, which could not be initiated because the patient's platelet count was below 50 × 10^9^/l with Hb 12,8 g/dl, WBC 9.11 × 10^9^/L, and neutrophils 7.8 × 10^9^/L. We decided to ask for an off-label use of Eltrombopag to increase platelet counts to safer levels for anticoagulation. The patient's written informed consent was requested. Eltrombopag, at an initial dose of 50 mg daily, was given, and, after an initial platelet recovery to 120 × 10^9^/l, Acenocoumarol treatment was started. After twelve months of treatment with Eltrombopag, the patient persisted with adequate platelet count (80 × 10^9^/L), allowing continued treatment without complications and starting intermittent doses of oral Chlorambucil.

Eltrombopag treatment was well tolerated with no evidence of adverse effects related to its administration without thrombotic complications during the two years of continuous treatment. Finally, in October 2014, the patient died of respiratory complications due to pneumonia with pleural effusion.

**Figure 1:**
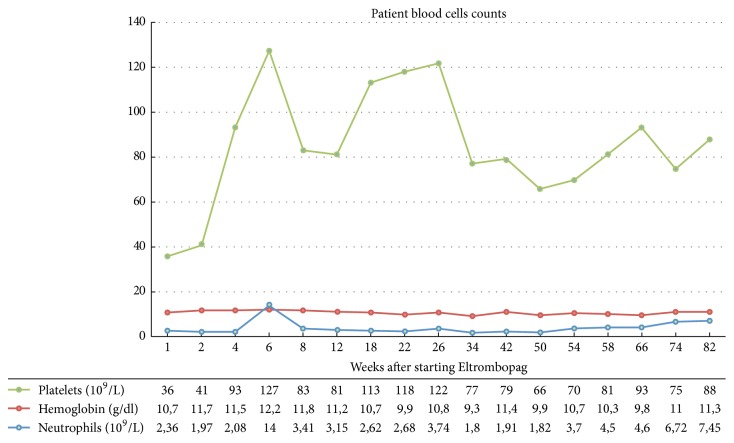
Patient platelet count after TPO-R agonist treatment.

We show (Figure 1) the evolution of the blood cell count under Eltrombopag treatment, with adequate drug response. The presence of CLL cells in blood or bone marrow appears to have no influence on drug efficacy.

## 3. Discussion

Up to 8% of patients with chronic lymphocytic leukemia (CLL), including those with stable diseases, develop secondary autoimmune disorders, which can confound cytopenias, complicate the course of their CLL, compromise their quality of life, and, in some cases, be fatal [10]. The most common of these autoimmune cytopenias is autoimmune hemolytic anemia (AIHA), which is an antibody-mediated destruction of autologous red blood cells (RBCs). The proportion of patients who present this pathology varies from 4.3 to 9.7% [3, 11, 12].

The second most common among autoimmune disorder associated with CLL is immune thrombocytopenic purpura (ITP), with an incidence of approximately 2–5% of the CLL population [3]. The ITP physiopathology is characterized by increased destruction and impaired production of platelets caused by autoantibodies directed against the platelets and megakaryocytes, making ITP diagnosis a clinical challenge.

Unlike AIHA, there are no ready markers for platelet destruction. Commercially available antiplatelet antibody testing is generally unhelpful and has not been expressly studied in CLL patients. CLL-associated thrombocytopenia may be due to bone marrow infiltration by the disease and the splenomegaly usually found in these patients. Thrombocytopenia in a patient with CLL can be considered immune-mediated when there is a sudden large fall in platelets in the absence of splenomegaly, infection, or chemotherapy and with plentiful megakaryocytes in the bone marrow [5].

Due to the risk of bleeding, it has been recommended to initiate therapy in those patients with platelet counts of <30 × 10^9^/L [13]. ITP conventional therapies are focused on preventing peripheral destruction of platelets and the impaired platelet production due to the direct effect on the bone marrow megakaryocytes mediated by autoantibodies.

In our case, we highlight the difficulty of treating the autoimmune manifestations associated with CLL. A platelet count > 30 × 10^9^/L and at least a twofold increase in the baseline count without evidence of bleeding can be considered as an ITP treatment good response [14]. Conventional treatment with corticosteroids and immunosuppression represents the first-line option for most patients; nevertheless, the most effective treatment is that directed at the underlying CLL [13]. Our patient did not respond to conventional therapy (corticosteroids or to intravenous immunoglobulins) or to a CLL-targeted chemoimmunotherapy regimen including Rituximab, during the search for new treatment alternatives, limited by his cardiovascular pathology history.

Although splenectomy continued to be an effective treatment, particularly for ITP, this procedure does not ensure a successful response. Provan et al. have reported success rates approach 90% with two-thirds of patients having a durable response [15]. And immunization and other prophylactic measures (as antibiotic therapy) will be required for this patients.

TPO receptor agonists (Romiplostim and Eltrombopag) represent a new option in the ITP treatment. These agents act by increasing the production of platelets rather than by avoiding their premature destruction. Both analogues have been reported to be successful in case reports of patients with CLL-associated ITP [7, 8, 16, 17]. Response to Romiplostim and Eltrombopag can be expected within 5–14 and 7–28 days, respectively [9]. Eltrombopag is an oral nonpeptide TPO receptor agonist that increases platelet production by binding to the transmembrane domain of the TPO receptor and inducing proliferation and differentiation of bone marrow progenitor cells in the megakaryocyte lineage [18]. Eltrombopag is approved by the Federal Drug Agency for chronic idiopathic ITP unresponsive to corticosteroids, intravenous immunoglobulins, or splenectomy. However, there is little published data on the use of TPO receptor agonists for ITP secondary to CLL.

The case, we report, illustrates the effectiveness of Eltrombopag in cases with refractory thrombocytopenia associated with CLL. The thrombopoietic mimetic agent allowed initiating and maintaining an oral anticoagulant treatment with Acenocoumarol that was indicated due to a severe arrhythmogenic cardiomyopathy. The platelet response was achieved quickly and platelet counts remained stable during 24 consecutive months under Eltrombopag therapy.

Other authors have communicated their experiences with the use of TPO mimetic drugs during short periods of time to increase platelet counts in patients with ITP before surgical procedures, to optimize the procedure and to reduce bleeding episodes. Our case illustrates how TPO mimetic drugs can be safely administered over long periods of time, helping to keep platelet counts above life-threatening levels and preserving the immune system because they are nonimmunosuppressive drugs.

The possibility of administering TPO mimetic drugs in severely thrombocytopenic patients who need to initiate or maintain an anticoagulant therapy is of great interest. Our clinical experience demonstrates its feasibility in patients with ITP associated with CLL.
